# Understanding the Exchange Interaction between Paramagnetic Metal Ions and Radical Ligands: DFT and Ab Initio Study on Semiquinonato Cu(II) Complexes

**DOI:** 10.3390/ijms24044001

**Published:** 2023-02-16

**Authors:** Aleksandra Ziółkowska, Maciej Witwicki

**Affiliations:** 1 Faculty of Chemistry, Wroclaw University of Science and Technology, Wybrzeże Wyspiańskiego 27, 50-370 Wroclaw, Poland; 2 Faculty of Chemistry, Wroclaw University, F. Joliot-Curie 14, 50-283 Wroclaw, Poland

**Keywords:** radicals, semiquinone, exchange coupling, DFT, ab initio, ferromagnetism, antiferromagnetism, broken symmetry

## Abstract

The exchange coupling, represented by the *J* parameter, is of tremendous importance in understanding the reactivity and magnetic behavior of open-shell molecular systems. In the past, it was the subject of theoretical investigations, but these studies are mostly limited to the interaction between metallic centers. The exchange coupling between paramagnetic metal ions and radical ligands has hitherto received scant attention in theoretical studies, and thus the understanding of the factors governing this interaction is lacking. In this paper, we use DFT, CASSCF, CASSCF/NEVPT2, and DDCI3 methods to provide insight into exchange interaction in semiquinonato copper(II) complexes. Our primary objective is to identify structural features that affect this magnetic interaction. We demonstrate that the magnetic character of Cu(II)-semiquinone complexes are mainly determined by the relative position of the semiquinone ligand to the Cu(II) ion. The results can support the experimental interpretation of magnetic data for similar systems and can be used for the in-silico design of magnetic complexes with radical ligands.

## 1. Introduction

Transition-metal complexes containing redox-active, proradical ligands have long been attracting the researcher’s attention because the distribution of electrons between the metal centers and such ligands provides unique electronic properties [1,2,3,4,5,6,7,8,9,10]. Extensive research has shown that the interplay of redox-active transition metal ions and pro-radical ligands plays an important role in biochemistry [11,12,13], with copper-containing enzymes being a prominent example [14,15,16]. For the copper complexes with catecholate ligands, which are an intermediate in the catalytic oxidation of catechols [17,18], the formation of semiquinonato copper complexes is a crucial step. These natural mechanisms inspired the design of catalytic systems with redox-active/radical ligands, including semiquinones [14,19,20,21,22].

Radical ligands also represent a growing field for another reason. They are increasingly important in the design of molecular materials that exhibit magnetic properties. Most ligands mediate antiferromagnetic interactions, but organic spin carriers can link metal centers providing high-spin molecules due to ferromagnetic or ferrimagnetic ordering [23,24,25,26]. Importantly, the exchange coupling between paramagnetic metal ions and radical ligands is usually much stronger than the superexchange interactions. The strength of exchange coupling is one of the critical parameters for single-molecule magnets. It determines the separation between the spin ground state and the spin excited states, which must be sufficiently large to maintain slow magnetization dynamics at elevated temperatures and shuts down fast quantum relaxation pathways. Therefore, it is hardly surprising that radical ligands are an important building block for such systems [27,28].

Another significant aspect of the interaction between transient metal ions and radicals is valence tautomerism, which is the ability to possess two or more isomeric forms that are connected by intramolecular electron transfer between the ligand and the metal [29,30,31,32,33]. Therefore, this feature can be applied, for instance, as a spin crossover to build magnetic switches [33], but it also occurs in enzymatic centers and tailors their reactivity [34]. 

**Figure 1:**
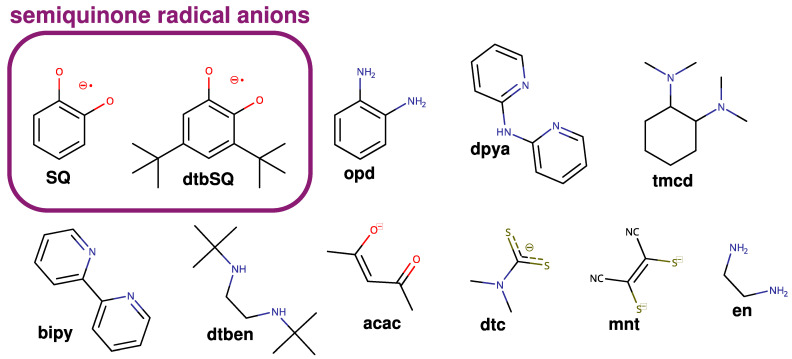
Ligands were used in this study.

It is now well established that theoretical methods are an efficient multipurpose tool for chemistry, biochemistry, and material science. Recent scientific efforts have shown that they can help us understand various phenomena, for example, reaction mechanisms or reactivity [35,36,37,38], electron density distribution [39,40,41], and elusive structure of complex biochemical systems or radical centers [42,43,44], anticancer [35,45] and antioxidant properties [46,47,48,49,50,51], soot formation [52] or pesticide decomposition [53]. The exchange coupling between paramagnetic centers has been a subject of such investigations at the density functional theory (DFT) and ab initio levels, but these studies were mainly limited to the interaction between metallic centers [54,55,56,57,58,59,60,61,62,63,64,65,66,67,68,69,70,71,72,73,74,75,76]. Theoretical studies on the exchange coupling between radical ligands and paramagnetic metal ions were performed [77,78,79,80,81]. However, in our opinion, a systematic understanding of how various structural and electronic features of such complexes affect exchange coupling is still lacking. It is surprising since exchange coupling is crucial for both magnetic properties and reactivity [82,83,84]. Therefore, this article discusses the case of semiquinonato copper(II) complexes, which have been studied experimentally for 40 years [18,85,86,87,88,89,90,91,92,93,94,95]. Our primary aim was to identify structural features that affect exchange coupling; however, a comparison with experimental data is also provided. In this investigation, we used broken-symmetry (BS) DFT and ab initio methods (CASSCF, CASSCF/NEVPT2, and DDCI3). The structures of all ligands used in this study are shown in Figure 1. 

The d^9^ Cu(II) ion and semiquinonanto ligand have local spins *S_A_* and *S_B_* with *S_A_* = *S_B_* = ½. Their magnetic coupling leads to two states with total spin *S* = 1 or 0. The order and spacing of these two states can be modeled by the isotropic Heisenberg-Dirac-Van Vleck Hamiltonian:

(1) $${\hat{H}}_{HDV}=-2J{\hat{S}}_{A}{\hat{S}}_{B}$$

where *J* is the exchange coupling constant that parametrizes the fictitious exchange interaction. It is positive for ferromagnetic (*S* = 1) and negative for antiferromagnetic (*S* = 0) coupling. According to the above Hamiltonian, the two spin states are spaced by 2*J*. All the experimental values of *J* used in this work were converted to fit this Hamiltonian.

## 2. Results and Discussion

### 2.1. General Considerations

**Figure 2:**
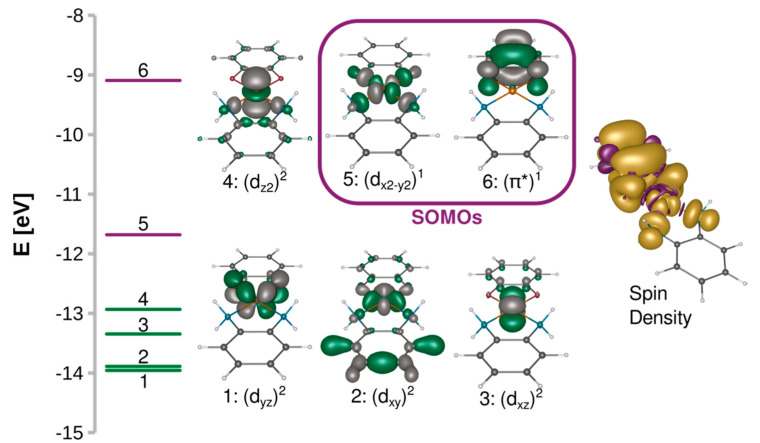
Splitting of the quasi-restricted orbitals and their isosurfaces contoured at 0.05 a.u. and spin density isosurfaces contoured at 0.001 a.u. calculated at the B3LYP/def2-TZVP level for **[Cu(SQ)(opd)]^+^**.

**Figure 3:**
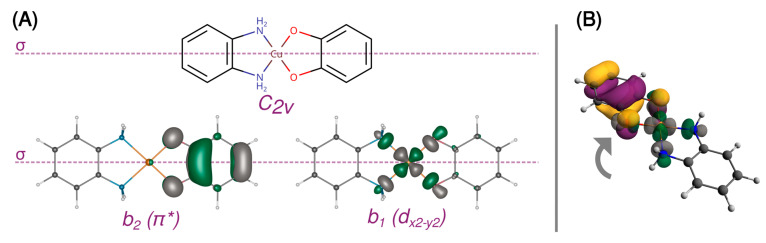
Orthogonal magnetic orbitals in **[Cu(SQ)(opd)]^+^** with the mirror plane σ (**A**) and the ineffective overlap between them (**B**). The isosurfaces were contoured at 0.05 a.u.

A 3d^9^ Cu(II) ion in the octahedral ligand field is subject to the Jahn–Teller distortion, which removes the degeneracy of the e_g_ and t_2g_ orbitals by a decrease in molecular symmetry. Typically, the 3d^9^ system becomes then an elongated octahedral, square pyramidal, or square planar complex with the unpaired electron occupying the molecular orbital with a strong d_x2−y2_ contribution. Figure 2 shows such split d orbitals for a model *C_2v_* square planar complex (**[Cu(SQ)(opd)]^+^**). The singly occupied (magnetic) molecular orbital (SOMO) of the copper(II) ion points along the Cu-N and Cu-O bonds in the basal plane. As shown in Figure 3A, it is antisymmetric with respect to the mirror plane *σ*, transforms as *b*_1_ in the *C_2v_* point group and is strongly *σ*-antibonding with respect to both ligands. When the CuN_2_O_2_ unit is planar, the two SOMOs are strictly orthogonal. The SOMO of Cu(II) is significantly delocalized toward the oxygen atoms of the o-semiquinone radical, whose SOMO has a significant density on the same oxygen atoms. Due to symmetry, the overlap density between two SOMOs has two equivalent positive and two negative lobes around each oxygen atom of the o-semiquinone (Figure 3B). Therefore, as explained by Kahn et al. [88], in a planar semiquinonato copper(II) complex, the total overlap between the two SOMOs should be zero, and the magnetic exchange interaction is expected to be ferromagnetic. Our BS B3LYP/def2-TZVP calculations for the model square planar **[Cu(SQ)(opd)]^+^** complex confirm that the overlap integral is zero and the magnetic exchange coupling is ferromagnetic with a large singlet-triplet energy gap (*J* = 294 cm^−1^). The magnitude of the ferromagnetic coupling for this model agrees with the experimental observation for **[Cu(dtbSQ)(dpya)(ClO_4_)]** (140 cm^−1^), **[Cu(dtbSQ)(bipy)(BF_4_)]** (191 cm^−1^) and **[Cu(dtbSQ)(dpya)(THF)_2_]^+^** (168 cm^−1^) [85].

### 2.2. Performance of Computational Methods

Before performing a detailed analysis, it is reasonable to compare the performance of functionals, ab initio methods, and basis sets. Table 1 presents the values of the magnetic exchange coupling *J* predicted for various model complexes. The results of calculations for the models with various ligands in the Cu(II) axial position are given in Table S1.

BS DFT calculations can be accurate when compared to the experimentally determined *J* values, but selecting the XC functional is a delicate matter. The GGA functionals overestimate exchange coupling [54,55,56,57,58,59,60], and for this reason, we did not include them in this investigation. There are known cases in which the physical interpretation of *J* depends on the chosen XC functional, but, favorably, all the functionals we tested predicted qualitatively similar *J* values that reflect strong ferromagnetic interactions. In general, the functional B3LYP and TPSSh predict larger *J* constants in comparison with PBE0. For most molecular models, the results obtained by the three hybrid functionals are spread over a narrow range of 25 cm^−1^. For instance, the predicted values for **[Cu(SQ)(opd)]^+^** at the B3LYP/def2-TZVP, TPSSh/def-TZVP and PBE0/def2-TZVP levels are 294, 276 and 271 cm^−1^, respectively. We observed the smallest differences of a few cm^−1^ for **[Cu(SQ)(dtc)]** and the largest, up to 70 cm^−1^, for the two model complexes with the **opd** ligand occupying the equatorial position and methanoate (**[Cu(SQ)(opd)(HCOO)]**) or chloride ion (**[Cu(SQ)(opd)(Cl)]**) in the axial position (Table S1).

The larger differences between the exchange coupling constants *J* should not be associated with negatively charged ligands occupying the axial position of Cu(II), as they are not observed for the complexes with tetrafluoroborate (**[Cu(SQ)(opd)(BF_4_)]**) and perchlorate anion (**[Cu(SQ)(opd)(ClO_4_)]**). Also, there is no evidence that the sulfur donor atoms reduce the spread, as indicated by the **[Cu(SQ)(dtc)]** model, as we did not observe this for **[Cu(SQ)(mnt)]^–^**.

Multireference calculations allow direct access to all spin states without the need for spin projection approximations. The CASSCF calculations with the minimal active space composed of two electrons in two SOMOs, labeled as CASSCF(2e,2o), predict ferromagnetic coupling but with a magnitude even over four times lower than the DFT methods. For example, the predicted *J* values at the B3LYP/def2-TZVP and CASSCF(2e,2o)/def2-TZVP theory level for **[Cu(SQ)(opd)]^+^** are 274 and 74 cm^−1^, respectively. 

An accepted strategy to improve reference wave functions and, thus, the magnetic couplings is to extend the minimal active space. Therefore, it was extended by the remaining 3d orbitals of copper(II) [CASSCF(10e,6o)], but the predicted *J* values remained unchanged. The active space was further expanded by four lone pairs that can undergo a bonding interaction with Cu(II) (these orbitals are shown in Figure S1). However, these CASSCF(18e,10o) calculations produced the *J* parameters comparable to those of CASSCF(2e,2o) and CASSCF(10e,6o). It becomes apparent that the CASSCF calculations with the minimal and extended active spaces are insufficient to quantitatively reproduce the magnitude of the exchange coupling observed experimentally. This underestimation of the ferromagnetic interaction can be attributed to the inability of the relatively small active space to capture the physics of the spin coupling and the inability of the method to recover dynamic electron correlation. These results reflect earlier observations for the antiferromagnetically coupled transition metal dimers [75,96,97].

To improve the methodology systematically, we included the dynamical correlation with the multiconfigurational reference perturbation (MRPT) using the n-electron valence state perturbation theory (NEVPT2) approach. Two different formulations of NEVPT2 were tested, that is, strongly contracted (SC-NEVPT2) and fully internally contracted NEVPT2 (FIC-NEVPT2). It is apparent from Table 1 that, regardless of the variant, NEVPT2 has a very limited effect on the predicted strength of the ferromagnetic coupling if applied to the CASSCF(2e,2o) wave function. This is clear in the case of **[Cu(SQ)(opd)]^+^**, for which the *J* parameter decreases by 1 cm^−1^ after applying the NEVPT2 correction. These results are in line with previous observations that the treatment of the electron correlation by the MRPT methods is limited in the minimal active space, and this leads to a systematic underestimation of the magnetic coupling [85,87].

The ferromagnetic coupling predicted by the NEVPT2 method for the CASSCF(10e,6o) wave function underwent a noticeable increase, especially well pronounced for **[Cu(SQ)(dtc)]**. The inclusion of the four lone-pair ligand orbitals in the active space [CASSCF(18e,10o)] was accompanied by a more significant increase in the ferromagnetic coupling. The predicted *J* values became closer to their experimental counterparts reported for **[Cu(dtbSQ)(dpya)(ClO_4_)]** (140 cm^−1^), **[Cu(dtbSQ)(bipy)(BF_4_)]** (191 cm^−1^) and **[Cu(dtbSQ)(dpya)(THF)_2_]^+^** (161 cm^−1^). The SC-NEVPT2 and FIC-NEVPT2 variants produced very similar numerical results, but for the largest active space we used, these differences were slightly more pronounced, and FIC-NEVPT2 tended to predict slightly stronger ferromagnetic couplings in comparison with SC-NEVPT2.

As suggested by previous studies on the exchange interaction between paramagnetic metal ions [61,62,63,64,65,66,96], the potentially very accurate singlet-triplet energy gap, and thus the *J* constant, is obtained with the difference dedicated MRCI (DDCI3) even with the minimal active space. This computational technique, in concert with the def2-TZVP basis set, produces *J* = 173 cm^−1^ for the model complex **[Cu(SQ)(opd)]^+^**, and this prediction is in line with the values 140, 191 and 161 cm^−1^ that were experimentally determined for structurally similar **[Cu(dtbSQ)(dpya)(ClO_4_)]**, **[Cu(dtbSQ)(bipy)(BF_4_)]** and **[Cu(dtbSQ)(dpya)(THF)_2_]^+^**, respectively [85]. The difference in accuracy between the result obtained at the minimal active space CASSCF(2e,2o)/NEVPT2 and DDCI3(2e,2o) calculations requires a brief comment. Regardless of the variant, NEVPT2 provides a perturbational estimate of the electron correlation effect beyond the minimal active space description. In contrast, DDCI accounts for electron correlation in a variational and, therefore, in principle, more precise manner. This has been discussed in detail by Malrieu et al. in their excellent review paper [96]. Regrettably, the DDCI3 calculations with extended active spaces were computationally too expensive to perform.

The choice of basis set can be important for the exchange coupling calculation. Selecting a fairly large basis set for paramagnetic metal ions while keeping reasonable ones for the other atoms is frequently recommended [69,98,99]. This has been shown to work well for systems without radical ligands. However, such an approach may lead to an unbalanced description of the spin density of radical ligand, and thus we used an equally accurate basis set on all atoms that allowed for carrying out the DDCI3(2e,2o) calculations for experimentally relevant models. Regardless of the method, the computationally efficient def2-SVP provides values noticeably, that is, up to 20%, different from def2-TZVP. In contrast, the cc-pVDZ and def2-TZVP basis sets produce quite similar results, especially in concert with the DDCI3(2e,2o) method. A good performance of cc-pVDZ in the *J* computations at the DDCI3(2e,2o) level was previously observed for the Cu(II) dinuclear complex with 4-N-(2′-pyridylimine)benzoic acid ligands [61].

### 2.3. Magnetostructural Correlations

In experimental studies, it is not straightforward to separate the effects of individual structural parameters on the exchange coupling constant. Conversely, molecular modeling offers the possibility of scanning *J* separately along the selected structural parameter. This was recently well demonstrated for the Mn(III) dinuclear complex [98].

To provide a detailed analysis of structural effects, we carried out a decomposition of the magnetic exchange coupling into its different physical mechanisms using BS DFT. For two *S* = ½ magnetic centers, the most important ones are the direct exchange (*J*_0_) and the kinetic exchange (Δ*J_KE_*) between the magnetic electrons and the spin (or core) polarization of the nonmagnetic electrons (Δ*J_CP_*):

*J* = *J*_0_ + Δ*J_KE_* + Δ*J_CP_* (2)

This decomposition starts with the high-spin state (HS) calculated in the restricted open-shell formalism (RO). The direct exchange contribution *J*_0_ is calculated by flipping the spin of one magnetic orbital and the immediate calculation of the BS energy with all the orbitals frozen (without the SCF procedure). Then, only the magnetic orbitals are relaxed in the field of the frozen nonmagnetic orbitals, allowing the magnetic orbitals to delocalize from one center to the other. From this procedure, the kinetic exchange Δ*J_KE_* is obtained. Finally, to calculate the core polarization contribution, the core orbitals are relaxed in both the HS RO and unrestricted BS determinants, keeping the magnetic orbitals frozen. The details are well described in the literature [67,68,69]; the Δ*J_CP_* contribution was calculated according to ref. [68]. Regarding this decomposition, some limitations need to be acknowledged. In this step-by-step procedure, we start from the HS RO formalism, and all orbitals are never relaxed in the same SCF procedure. Thus, the final orbitals can slightly differ from the ones generated in the "standard" BS calculation. Therefore, the *J* parameter predicted according to Equation (2) can slightly deviate from the results of the standard BS calculation. A similar decomposition was also done with high-level quantum-mechanical methods by selectively considering classes of excitations in configuration interaction calculations [70], but the computational cost limits its applicability to small molecules.

For the optimized **[Cu(SQ)(opd)]^+^** model, we found at the B3LYP/def2-TZVP theory level that *J*_0_ = 244 cm^−1^, Δ*J_KE_* = −1 cm^−1^ and Δ*J_CP_* = 22 cm^−1^. The insignificant Δ*J_KE_* contribution is consistent with the orthogonality of the magnetic orbitals *d*_*x*2−*y*2_ and π* discussed in Section 2.1 General Considerations. The quality of the decomposition can be assessed by comparing the total *J* value calculated according to Equation (2) with the value produced by the standard BS calculation. The sum of the three contributions is approximately 10% lower than the result of the standard BS B3LYP/def-TZVP calculations. This, along with the insignificant Δ*J_KE_* contribution, shows that the adapted decomposition scheme is reasonable. An interesting thing to note is that, for the model **[Cu(SQ)(opd)]^+^**, the spin polarization supports the ferromagnetic coupling (Δ*J_CP_ > 0*). The magnitude of Δ*J_CP_* may seem low, but for the Cu(II) dinuclear complexes that exhibit significant magnetic exchange, this contribution was between –1 and +9 cm^−1^ [69].

**Figure 4:**
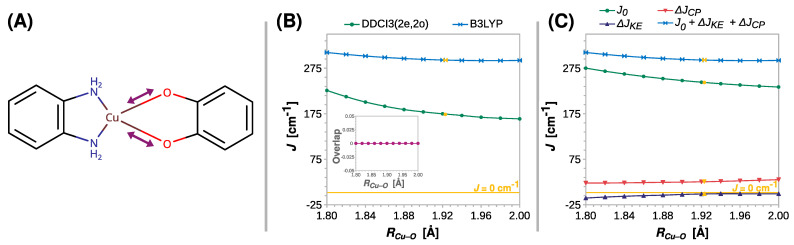
Rigid scan of *J* along the Cu–O distance (*R_Cu–O_*): schematic representation of the varied structural parameter (**A**); the parameter *J* predicted at the B3LYP and DDCI3(2e,2o) levels (**B**); decomposition of *J* at the B3LYP level (**C**). Inset in (**B**) shows the overlap of the magnetic orbitals calculated at the B3LYP level. All are calculated with the def2-TZVP basis set. For clarity, the line *J* = 0 cm^−1^ is shown as a reference. For the geometry optimized at the B3LYP/def2-TZVP theory level *R_Cu–O_* = 1.925 Å (labeled with the yellow symbols).

The length of the coordination bond is an important parameter that influences the properties of the coordination compounds. This inspired a search for the correlation between the exchange coupling constant *J* and the Cu–O distance (*R_Cu–O_*). The results of the appropriate rigid scan are shown in Figure 4. Surprisingly, only a minor decrease in *J* was predicted by DFT with increasing *R_Cu–O_*. The value of *J* fell from 310 to 292 cm^−1^ at the B3LYP theory level, but the weakening of the ferromagnetic coupling was more substantial when the DDCI3(2e,2o) approach was used. This ab initio method predicted a reduction in *J* by more than a fourth, that is, from 226 to 164 cm^−1^, strongly suggesting that *R_Cu–O_* affects the coupling strength in semiquinonato Cu(II) complexes. The results of the scans performed at the CASSCF(2e,2o) and CASSCF(2e,2o)/NEVPT2 levels are shown in Figures S2–S5.

Although the DFT methods underestimate the role of this structural parameter, the decomposition of *J* at the B3LYP level qualitatively revealed the reasons for the decrease in the ferromagnetic coupling. What stands out in Figure 4C is that the change in kinetic exchange Δ*J_KE_* with *R_Cu–O_* is negligible, and the reduction in the ferromagnetic coupling stems from the decrease in direct exchange *J*_0_, which is, however, alleviated by the increasing Δ*J_CP_* contribution.

**Figure 5:**
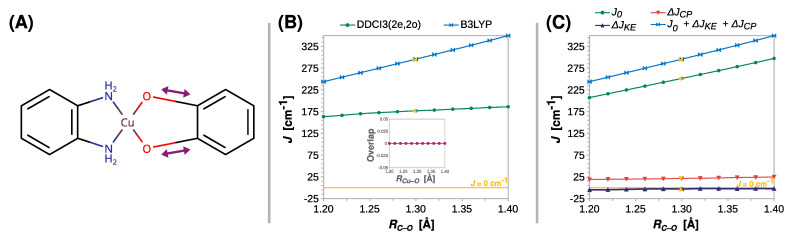
Rigid scan of *J* along the C–O distance (*R_C–O_*): schematic representation of the varied structural parameter (**A**); the parameter *J* predicted at the B3LYP and DDCI3(2e,2o) levels and DDCI3(2e,2o) (**B**); decomposition of *J* at the B3LYP level (**C**). Inset in (**B**) shows the overlap of the magnetic orbitals calculated at the B3LYP level. All are calculated with the def2-TZVP basis set. For clarity, the line *J* = 0 cm^−1^ is shown as a reference. For the geometry optimized at the B3LYP/def2-TZVP theory level, *R_C–O_* = 1.294 Å (labeled with the yellow symbols).

As shown previously in the studies on the EPR *g* tensor, the length of the C*–*O bond (*R_Cu–O_*) can have a significant impact on the properties of semiquinone radicals [100,101] and, as shown in Figure 5, this is also true for the strength of exchange coupling in their Cu(II) complexes. Contrary to *R_Cu–O_*, the functional B3LYP and DDCI3(2e,2o) showed that *J* increases as the C*–*O bond elongates. Compared to DDCI3(2e,2o), the functional B3LYP yielded a more significant rise in the *J* value. The breakdown of *J* at the B3LYP level exposed that the contribution of the direct exchange *J*_0_ dominates the observed increase, as Δ*J_KE_* remains insignificant and Δ*J_CP_* increases only slightly, that is, from 19 to 24 cm^−1^ (Figure 5C).

**Figure 6:**
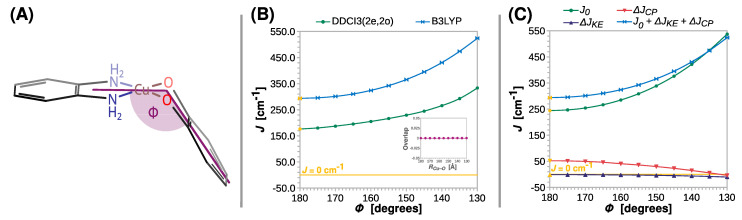
Rigid scan of *J* along the bending angle between the N-Cu-N and O-Cu-O planes (*Φ*): schematic representation of the varied structural parameter (**A**); the parameter *J* predicted at the B3LYP and DDCI3(2e,2o) levels (**B**); decomposition of *J* at the B3LYP level (**C**). Inset in (**B**) shows the overlap of the magnetic orbitals calculated at the B3LYP level. All are calculated with the def2-TZVP basis set. For clarity, the line *J* = 0 cm^−1^ is shown as a reference. For the geometry optimized at the B3LYP/def2-TZVP theory level *Φ* = 0° (labeled with the yellow symbols).

For the optimized model **[Cu(SQ)(opd)]^+^**, two ligands are coplanar, but, as shown by the X-ray diffraction experiments [18,54,85,102,103], this is rarely the case. Therefore, the impact of divergence from coplanarity should be investigated. Let us start with the bending angle (*Φ*), which is shown schematically in Figure 6A. 

Regardless of the computational method, the strength of ferromagnetic coupling increases gradually with *Φ*, which is reflected in the value of *J* going up from 294 to 524 cm^−1^ and from 176 to 334 cm^−1^ at the B3LYP and DDCI3(2e,2o) level, respectively. As for *R_Cu–O_* and *R_C–O_*, the two magnetic orbitals stay orthogonal, and therefore the Δ*J_KE_* contribution remains insignificant; the change in *J* is dominated by the direct exchange *J*_0_. Interestingly, Δ*J_CP_* steadily decreases to a low point of approximately −2 cm^−1^ at *Φ* = 130° (a small antiferromagnetic contribution to *J*).

**Figure 7:**
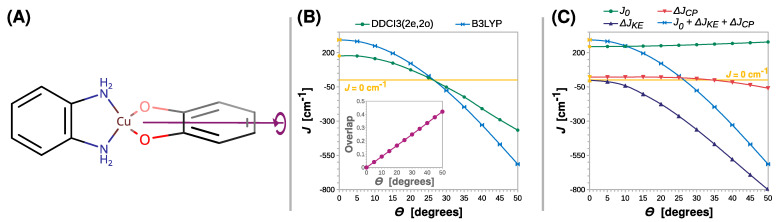
Rigid scan of *J* along the twisting angle between the N-Cu-N and O-Cu-O planes (*Θ*): schematic representation of the varied structural parameter (**A**); the parameter *J* predicted at the B3LYP and DDCI3(2e,2o) levels (**B**); decomposition of *J* at the B3LYP level (**C**). Inset in (**B**) shows the overlap of the magnetic orbitals calculated at the B3LYP level. All are calculated with the def2-TZVP basis set. For clarity, the line *J* = 0 cm^−1^ is shown as a reference. For the geometry optimized at the B3LYP/def2-TZVP theory level *Θ* = 0° (labeled with the yellow symbols).

Next, we monitored the rhombic distortion of the Cu(II) coordination sphere. This was carried out by the rigid scan in which the intersection angle *Θ* between the N-Cu-N and O-Cu-O planes was varied. The twist of the **SQ** ligand out of the basal plane is schematically shown in Figure 7A. What is striking about Figure 7B is that, conversely to previously analyzed distortions, the twist of the radical ligand can clearly change the nature of magnetic coupling into antiferromagnetic (the *J* parameter becomes negative). The B3LYP and DDCI3(2e,2o) calculations for the simple model **[Cu(SQ)(opd)]^+^** suggest that the crossover point lies at an angle of approximately 25°. 

The change in the nature of magnetic interaction in the semiquinonato Cu(II) complexes can be triggered by the twist of the radical ligand and can be explained by the orthogonality of the copper *d_x2-y2_* and the semiquinone π* magnetic orbitals. The elongation of the Cu–O or C–O bonds and the increase in bending angle *Θ* preserve the mirror plane *σ*, which is shown in Figure 3A. Therefore, as shown in the insets in Figure 4B, Figure 5B and Figure 6B, the overlap integral between the two magnetic orbitals remains zero; they stay orthogonal and promote the ferromagnetic coupling. However, upon the rhombic distortion, the minor plane *σ* vanishes, and the overlap integral between the two magnetic orbitals becomes non-zero and stabilizes the antiferromagnetic state. This is because the overlap improves bonding in the antiferromagnetic state and destabilizes the triplet state because of the Pauli repulsion between two unpaired electrons. The inset in Figure 7B shows that the overlap integral between the magnetic orbitals increases linearly with *Θ*.

A wider understanding of the crossover between ferro- and antiferromagnetic coupling in the semiquinonato Cu(II) complexes can be developed by the decomposition of *J* into the three contributions. The most important finding of this analysis is that with the increase in *Θ*, the kinetic exchange Δ*J_KE_* rapidly becomes negative and cancels the positive contribution of the direct exchange *J*_0_. After the crossover point, Δ*J_KE_* dominates the magnetic coupling. The interesting feature is that the twist of the radical ligand increases *J*_0_ by 34 cm^−1^, but this effect can only slightly alleviate rising Δ*J_KE_*. Furthermore, the rhombic distortion influence Δ*J_CP_*. The ferromagnetic contribution from the core polarization decreases, and starting from *Θ* = 35°, it becomes negative and thus stabilizes the antiferromagnetic state.

### 2.4. Comparison with Experiment

**Figure 8:**
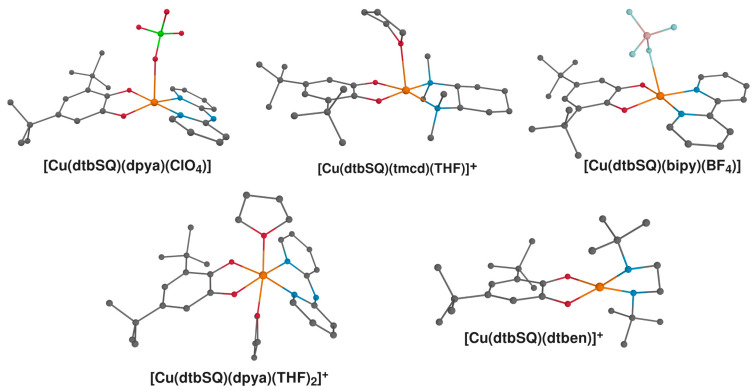
Structures of semiquinonato Cu(II) complexes characterized experimentally that were included in this study: **[Cu(dtbSQ)(dpya)(ClO_4_)]** [87], **[Cu(dtbSQ)(tmcd)(THF)]** [18], **[Cu(dtbSQ)(tmcd)(THF)]** [85]; **[Cu(dtbSQ)(bipy)(BF_4_)]** [85], **[Cu(dtbSQ)(dpya)(THF)_2_]^+^** [85] and **[Cu(dtbSQ)(dtben)]^+^** [18]. Hydrogen atoms were omitted for clarity; Cu: orange; C: grey; N: blue; O: red; B: pale pink; Cl: green; F: pale blue.

Rather than aiming at reproducing experimental values, in the present study, our goal was to provide a reference set of correlations between structural parameters and magnetic properties in semiquinonato Cu(II) complexes. In doing so, we emphasized obtaining qualitative insight through the examination of the mechanisms of magnetic coupling, particularly as revealed through the decomposition of *J* into *J*_0_, Δ*J_KE_* and Δ*J_CP_*. Nevertheless, a comparison with the experimental results is always reasonable to perform, even if the environmental effects are neglected in the computations. The results of the calculations carried out for the systems that have been realized synthetically [18,85] are given in Table 2, and the molecular units used in these calculations are shown in Figure 8. To avoid introducing additional errors or bias in our comparison of methods, the counterions or solvent molecules were not removed if they occupied the axial position. Ferromagnetic coupling is an exceedingly common situation in complexes comprising Cu(II) and semiquinone radicals; therefore, only one complex represents a case of antiferromagnetic coupling, that is, **[Cu(dtbSQ)(dtben)]^+^.**

As seen in Table 2, for all experimental complexes, the DFT methods overestimate the strength of magnetic coupling but correctly predict the sign of *J*. Regardless of the functional, for the complex **[Cu(dtbSQ)(dpya)(ClO_4_)]**, the *J* is overestimated more than twice compared to the experimental counterpart. The agreement between DFT and experiment is significantly better for **[Cu(dtbSQ)(bipy)(BF_4_)]**, **[Cu(dtbSQ)(dpya)(THF)_2_]^+^** and antiferromagnetically coupled **[Cu(dtbSQ)(dtben)]^+^**, but the overestimation is still noticeable. Among the functionals tested, PBE0 provides results that are closer to the experiment, but it is not a significant improvement over B3LYP or TPSSh. The *J* values computed at the DFT level using the def2-TZVP are superior to those provided by def2-SVP, but the differences between the def2-TZVP and cc-pVDZ basis set are less apparent.

In contrast to DFT and regardless of the active space size, the CASSCF approach produced *J* parameters that are underestimated noticeably in comparison with experimentally determined values. For the minimal (2e,2o) active space, the SC- and FIC-NEVPT2 methods did not correct the predicted *J* parameter, except for the case of antiferromagnetic coupling for **[Cu(dtbSQ)(dtben)]^+^**. This is consistent with the results of the rigid scan along the twist angle *Θ* at the CASSCF(2e,2o)/SC-NEVPT2 theory level (Figure S5), which confirmed that even with the minimal active space, the perturbative correction provides significant improvement when the magnetic coupling between the radical ligand and Cu(II) ion becomes antiferromagnetic. 

For the ferromagnetically coupled complexes, the extension of the active space to (10e,6o) did not have a significant impact on the results of the NEVPT2 calculations. However, the inclusion of the four lone pairs in the active space [(18e,10o)] clearly increased the predicted strength of the ferromagnetic coupling and thus improved the agreement between theoretical and experimental *J* values. The agreement was very good for **[Cu(dtbSQ)(dpya)(ClO_4_)]** and good for **[Cu(dtbSQ)(dpya)(THF)_2_]^+^** if the basis cc-pVDZ was used in the calculations. The extension of the active space decreased the strength of the antiferromagnetic coupling for **[Cu(dtbSQ)(dtben)]^+^** (the *J* parameter became less negative) and thus slightly worsened the agreement between theory and experiment. This showed that the prediction of *J* at the CASSCF(2e,2o)/NEVPT2 level for **[Cu(dtbSQ)(dtben)]^+^** benefited from errors cancelations.

For the DDCI3(2e,2o) method, the computations with def2-TZVP become prohibitively expensive. However, our test calculations for smaller systems, which are summarized in Table 1 and discussed in Section 2.2 *Performance of Computational Methods*, showed that at this ab initio theory level, results obtained with def2-TZVP and cc-pVDZ should be comparable. Looking at Table 2, it is immediately apparent that the DDCI3(2e,2o)/cc-pVDZ calculations well reproduced the experimental values of *J*, with a somewhat larger discrepancy observed only for **[Cu(dtbSQ)(dpya)(ClO_4_)]**. This again shows that the variational approach to dynamic correlation at the DDCI(2e,2o) level is more accurate than the perturbational corrections for the CASSCF wave function.

The antiferromagnetically coupled complex **[Cu(dtbSQ)(dtben)]^+^** requires a short comment. Using the rigid scan approach, we have been able to demonstrate that the increase in the intersection angle *Θ* between the N-Cu-N and O-Cu-O planes can switch the magnetic coupling from ferromagnetic to antiferromagnetic, which can become significant for large *Θ*. Our conclusion from the scan of *J* along *Θ* stays in line with the strong antiferromagnetic coupling determined for **[Cu(dtbSQ)(dtben)]^+^** from the temperature dependence of the NMR chemical shifts [18]. For **[Cu(dtbSQ)(dtben)]^+^**, the intersection angle amounts to 51°, which reflects a strong rhombic distortion. Moreover, this ferromagnetic coupling was well reproduced in our computations for this complex, especially well at the DDCI3/ccpVD theory level.

## 3. Methods and Materials

We performed all computations using ORCA 4.2.1 [104,105]. Geometry optimizations were carried out at the DFT level with the hybrid functional B3LYP [106,107,108], def2-TZVP basis set [109] and the D3 dispersion corrections proposed by Grimme [110]. The accuracy of the DFT-optimized structures is generally excellent to good compared to X-ray diffraction data [54,102,103], although a systematic deviation is always expected compared to experimental data at finite temperatures [111]. Following previous practice, the high-spin state (HS) was assumed for optimizations [60,71,72]. For systems that have been realized synthetically, the structures were taken from the Cambridge Structural Database [112], and only the positions of hydrogen atoms were optimized after the noncoordinated counterions and solvent molecules were removed, and hydrogens were added where necessary. These structures are shown in Figure 8 with references in the description. All the optimized structures are given in the Supplementary Materials in XYZ format.

Subsequently, single-point calculations were performed to obtain the final energies of the S = 1 and S = 0 states. In these calculations, the basis sets def2-SVP [109], def2-TZVP [109] and correlation-consistent cc-pVDZ [75,96,97] were used. At the DFT level, we used the hybrid functional B3LYP [106,107,108], PBE0 [113] and TPSSh [114] and followed the standard broken symmetry (BS) DFT procedure using the FlipSpin feature of ORCA to generate the initial guess for the BS calculation. All BS solutions were verified for correctness by examining the spin populations to confirm the expected number of unpaired electrons at the Cu(II) and semiquinone sites. Exchange coupling constants *J* were computed with the Yamaguchi formula [115,116] that covers weak to strong exchange coupling by scaling the energy difference between the high-spin and broken-symmetry solutions according to their spin expectation values:

(3) $$J=\frac{E_{HS}-E_{BS}}{S^{2}{}_{HS}-S^{2}{}_{BS}}$$

The overlap integrals between two SOMOs were obtained at the BS B3LYP/def2-TZVP theory level by applying the corresponding orbital transformation [117].

Multireference CASSCF calculations were carried out in a state-averaged manner. For the minimal active space that describes singlet-triplet splitting, that is, two electrons in two SOMOs [CASSCF(2e,2o)], the initial orbitals were quasi-restricted DFT orbitals [118]. Two larger active spaces were tested. The first was constructed by adding the remaining four 3d orbitals of copper(II) [CASSCF(10e,6o)], and the second also included four lone pairs of ligands that can undergo bonding interaction with Cu(II) (these orbitals are shown in Figure S1) [CASSCF(18e,10o)]. The initial orbitals for CASSCF(10e,6o) and CASSCF(18e,10o) were obtained by the localization of the internal orbitals from CASSCF(2e,2o) using the Pipek−Mezey localization algorithm [119]. An overview of CASSCF can be found in ref. [120]. Two variants of the internally contracted N-electron valence state perturbation theory (NEVPT2) were adopted to apply the dynamical correlation to the CASSCF wave function, that is, strongly contracted NEVPT2 (SC-NEVPT2) and a fully internally contracted NEVPT2 (FIC-NEVPT2) [121,122,123] FIC-NEVPT2 is frequently referred to as partially contracted NEVPT2 (PC-NEVPT2).

The magnetic coupling constants *J* were also evaluated by means of difference dedicated configuration interaction with excitations up to three degrees of freedom (DDCI3). The DDCI3 approach is essentially MRCI with singles and doubles, where the two-hole two-particle excitations are omitted [124,125]. For the DDCI3 calculations, the CASSCF(2e,2o) reference wavefunctions were used with the same active space, and hence these calculations were referred to as DDCI3(2e,2o). This method with minimal active space was demonstrated to provide highly accurate *J* values for magnetic systems while remaining computationally feasible [61,96,125,126,127,128,129,130]. In the case of the ab initio methods, the value of *J* was calculated as follows:

(4) $$J=\frac{1}{2}\left(E_{S=1}-E_{S=0}\right)$$

For hybrid DFT, very tight energy convergence criteria (VeryTightSCF), enhanced integration grids (Grid6) and the chain-of-spheres approximation to exact exchange (COSX) [131] with increased grid settings (GridX6) were employed. The auxiliary def2/J basis sets were used in combination with def2-SVP and def2-TZVP, while for cc-pVDZ, the auxiliary basis set was generated using an automatic procedure (AutoAux). For the CASSCF calculations, we used the conventional integral storage and the RI approximation for the integral transformation and the Fock matrix construction (RIJK) to speed up the calculations [132,133]. Thus, the appropriate auxiliary basis sets for correlation calculations were employed, that is, def2/JK [134] for def2-SVP and def2-TZVP and the AutoAux procedure for cc-pVDZ. In the DDCI3 calculations, all single excitations were included, and the thresholds T_sel_ and T_pre_, defined elsewhere [135,136], were set to 10^−6^ and 10^−4^, respectively. These parameters can affect the computed *J* values, and thus they were validated (Tables S2–S4). As for CASSCF, the RI approximation for the integral transformation was used with the same correlation fitting basis sets.

We used Marvin for drawing chemical structures (version 21.2.0, ChemAxon https://www.chemaxon.com accessed on 15 February 2023). 3-D visualizations were done with the Gabedit 2.5.0 [137] (Figure 2, Figure 3A and Figure 8) and Avogadro 1.2 [138] (Figure 3B) software, both in combination with the PovRay ray-tracing program (version 3.7, http://www.povray.org/ accessed on 15 February 2023).

## 4. Conclusions

In this study, we theoretically analyzed the exchange coupling for a set of copper(II) complexes with semiquinone radicals. We used three hybrid DFT functionals, CASSCF with NEVPT2 for dynamical correlation and DDCI3. Rather than aiming at reproducing experimental values, in the present study, our objective was to uncover correlations between structural parameters and exchange coupling. We showed that the elongation of the Cu–O bonds and the rhombical distortion that twists the radical ligand decrease the ferromagnetic coupling, while the elongation of the C–O bond and bending of the radical ligand out of planarity increase it. From the inspected structural modifications, the twist of the radical ligand out of the basal plane deserves special attention. It can easily switch the magnetic interaction from ferromagnetic to antiferromagnetic and, thus, is the most suitable for tuning magnetic exchange couplings.

To provide more comprehensive insight into structural effects, we carried out a decomposition of the magnetic exchange coupling into three different contributions, that is, the direct exchange (*J*_0_), the kinetic exchange (Δ*J_KE_*) and the spin (or core) polarization of the nonmagnetic electrons (Δ*J_CP_*). The bending of the semiquinone ligand out of the complex planarity and the elongation of the Cu–O and C–O bonds correspond to the changes in *J*_0_. The twist of the radical ligand differed from these three modifications, as it enhanced Δ*J_KE_*, which is an antiferromagnetic contribution. We showed that, for semiquinonoato Cu(II) complexes, this structural modification and the increase in Δ*J_KE_* correlates with the vanishing orthogonality between two orbitals occupied by the unpaired electrons.

Although it was not the primary aim of our work, we conducted computations for the complexes that have been synthesized. All the methods tested correctly predict the ground spin state of the systems. The hybrid functionals overestimate, and CASSCF underestimates the values of *J*, even if the active space is extended to (18e,10o). We should note that for the complexes with ferromagnetic coupling between Cu(II) and semiquinone, the NEVPT2 correction noticeably improves the results if the calculations are done with relatively large (18e,10o) active space. In the case of antiferromagnetic coupling observed for **[Cu(dtbSQ)(dtben)]^+^**, the NEVPT2 correction improves the predicted *J* parameter even in combination with the minimal (2e,2o) active space. In summary, to obtain qualitative estimates of *J*, it is sufficient to perform DFT or CASSCF calculations with minimal active space with just the magnetic orbitals and the unpaired electrons. However, to obtain more quantitative numbers, one should add the remaining 3d and ligands lone pair orbitals to the active space and apply the NEVPT2 correction. However, the highly accurate predictions of *J* can be made at the DDCI3 level with minimal active space. For DFT methods, we found that PBE0 was slightly better than B3LYP and TPSSh.

We believe that our results improve the understanding of the exchange coupling between Cu(II) ions and semiquinone radicals and can be helpful in the experimental interpretation of magnetic data for such systems, and through generalization, they can be extended to other complexes of paramagnetic ions, with π-type radical ligands. The comprehensive analysis of the magnetic exchange we provided in this paper can be used for in silico design of molecular compounds with selected magnetic properties.

## Figures and Tables

**Table 1:** Exchange coupling constants *J* (in cm^−1^) calculated with DFT and ab initio methods for model semiquinonato Cu(II) complexes.

					CASSCF(2e,2o)				CASSCF(10e,6o)				CASSCF(18e,10o)		
	Basis Set	B3LYP	PBE0	TPSSh	without MRPT	SC-NEVPT2	FIC-NEVPT2	DDCI3(2,2)	without MRPT	SC-NEVPT2	FIC-NEVPT2		without MRPT	SC-NEVPT2	FIC-NEVPT2
**[Cu(SQ)(opd)]^+^**	def2-TZVP	294	271	276	74	73	73	173	75	81	80		76	130	129
	def2-SVP	339	316	311	85	88	88	193	85	93	92		85	124	130
	cc-pVDZ	298	276	282	79	77	77	169	79	85	84		80	135	137
**[Cu(SQ)(acac)]**	def2-TZVP	274	257	269	75	78	78	187	75	86	85		76	123	129
	def2-SVP	300	284	286	85	93	93	174	85	97	94		86	124	132
	cc-pVDZ	283	265	279	80	83	83	188	80	93	92		81	135	138
**[Cu(SQ)(dtc)]**	def2-TZVP	221	216	215	71	73	73	179	71	81	81		73	124	129
	def2-SVP	239	236	231	81	89	89	184	78	116	113		82	145	153
	cc-pVDZ	232	225	225	76	78	78	170	74	128	127		74	174	169
**[Cu(SQ)(en)]^+^**	def2-TZVP	292	269	274	74	72	72	185	74	75	74		74	106	117
	def2-SVP	337	314	310	85	88	88	199	85	92	92		86	128	135
	cc-pVDZ	297	274	279	78	76	76	180	79	109	108		78	175	177
**[Cu(SQ)(mnt)]^−^**	def2-TZVP	308	292	325	76	83	83	193	76	85	87		77	127	131
	def2-SVP	324	309	338	87	99	99	188	87	103	104		88	138	138
	cc-pVDZ	330	312	345	82	89	89	186	82	91	91		82	134	137

**Table 2:** Exchange coupling constants *J* (in cm^−1^) were calculated with DFT and ab initio methods for the semiquinonato Cu(II) complexes that were characterized experimentally.

					CASSCF(2e,2o)				CASSCF(10e,6o)				CASSCF(18e,10o)			Exptl.
	Basis Set	B3LYP	PBE0	TPSSh	without MRPT	SC-NEVPT2	FIC-NEVPT2	DDCI3(2,2)	without MRPT	SC-NEVPT2	FIC-NEVPT2		without MRPT	SC-NEVPT2	FIC-NEVPT2	
**[Cu(dtbSQ)(dpya)(ClO_4_)]**	def2-TZVP	297	274	295	72	73	74	-	75	87	85		74	100	117	140 ^(a)^
	def2-SVP	335	309	324	81	86	87	218	84	88	91		83	116	125	140 ^(a)^
	cc-pVDZ	312	301	311	77	78	79	204	82	86	85		81	125	130	140 ^(a)^
**[Cu(dtbSQ)(tmcd)(THF)]^+^**	def2-TZVP	241	227	229	69	66	71	-	74	73	74		73	108	114	FM ^(b)^
	def2-SVP	265	251	247	77	78	79	246	82	85	85		81	109	114	FM ^(b)^
	cc-pVDZ	250	234	237	73	70	71	210	77	80	79		77	122	124	FM ^(b)^
**[Cu(dtbSQ)(bipy)(BF_4_)]**	def2-TZVP	297	276	291	79	78	79	-	85	86	86		84	119	129	191 ^(a)^
	def2-SVP	335	312	321	88	91	92	215	92	95	95		90	120	127	191 ^(a)^
	cc-pVDZ	310	287	304	84	83	84	201	85	88	88		85	131	133	191 ^(a)^
**[Cu(dtbSQ)(dpya)(THF)_2_]^+^**	def2-TZVP	264	244	268	69	69	70	-	73	79	80		72	119	121	168 ^(a)^
	def2-SVP	295	273	284	77	81	81	210	82	85	87		80	106	112	168 ^(a)^
	cc-pVDZ	275	253	270	73	74	74	188	74	79	80		75	119	121	168 ^(a)^
**[Cu(dtbSQ)(dtben)]^+^**	def2-TZVP	−1589	−1312	−1788	−284	−560	−611	-	−296	−495	−519		−298	−501	−516	−1150 ^(c)^
	def2-SVP	−1790	−1489	−1933	−292	−548	−553	−809	−302	−493	−525		−303	−506	−519	−1150 ^(c)^
	cc-pVDZ	−1639	−1367	−1852	−299	−605	−655	−1140	−311	−582	−608		−312	−577	−593	−1150 ^(c)^

(a) [85]. (b) *FM* = (strong) ferromagnetic coupling [18]. (c) [18]

## Data Availability

The data presented in the current study are available in the article and in the associated Supplementary Material.

## Supplementary Materials

The following supporting information can be downloaded at: https://www.mdpi.com/article/10.3390/ijms24044001/s1.
