# Supplementary material for: Understanding the Exchange Interaction between Paramagnetic Metal Ions and Radical Ligands: DFT and Ab Initio Study on Semiquinonato Cu(II) Complexes
Source: Int J Mol Sci. 2023 Feb 16;24(4):4001. doi: 10.3390/ijms24044001 (PMC9959031; doi:10.3390/ijms24044001)
Supplement: Supplementary file 1 [file ijms-24-04001-s001.zip › ijms-2116148-supplementary.pdf]

Table S1. Exchange coupling constants *J* (in cm^{-1}) calculated with DFT and *ab initio* methods for model semiquinonato Cu(II) complexes with various ligands in the axial position.

					CASSCF(2,2)		
	basis set	***B3LYP***	***PBE0***	***TPSSh***	***No MRPT***	***SC-NEVPT2***	***DDCI3(2,2)***
**[Cu(SQ)(opd)(HCOO)]**	*def2-TZVP*	331	305	362	77	78	228
	def2-SVP	359	333	379	87	93	201
	cc-pVDZ	344	315	378	82	83	218
**[Cu(SQ)(opd)(Cl)]**	*def2-TZVP*	367	335	402	80	81	198
	def2-SVP	402	368	427	90	96	187
	cc-pVDZ	380	346	415	85	87	206
**[Cu(SQ)(opd)(NH_3)]$^+$**	*def2-TZVP*	330	305	335	74	75	190
	def2-SVP	369	341	363	84	90	187
	cc-pVDZ	343	316	348	79	81	196
**[Cu(SQ)(opd)(H_2O)]$^+$**	*def2-TZVP*	288	268	278	71	71	199
	def2-SVP	327	305	307	82	86	179
	cc-pVDZ	297	276	287	76	76	199
**[Cu(SQ)(opd)(ClO_4)]**	*def2-TZVP*	313	291	318	78	79	203
	def2-SVP	348	324	342	88	93	178
	cc-pVDZ	324	300	330	83	84	207
**[Cu(SQ)(opd)(THF)]$^+$**	*def2-TZVP*	301	278	298	73	73	233
	def2-SVP	335	311	323	82	87	191
	cc-pVDZ	310	287	308	78	78	212
**[Cu(SQ)(opd)(BF_4)]**	*def2-TZVP*	301	280	303	76	77	206
	def2-SVP	336	314	328	86	92	179
	cc-pVDZ	311	288	314	81	82	199

Effect of T_{pre} and T_{sel} on the value of *J* predicted at the DDCI3/def2-TZVP theory level. T_{pre} and T_{sel} are defined in:

1. Neese, F. J Chem Phys 2003, 119, 9428–9443, doi:10.1063/1.1615956.

2. Neese, F. Magnetic Resonance in Chemistry 2004, 42, S187–S198, doi:10.1002/mrc.1456.

There was no change in the predicted ΔE(S-T), and thus in the parameter *J*, if all singles excitations were included.

Table S2. Effect of T_{pre}; all single excitations were included and $T_{sel} = 10^{-6}$.

T_{pre}	ΔE(S-T) [cm^{-1}]	J [cm^{-1}]
10^{-2}	346	173
10^{-3}	346	173
10^{-4}	346	173
10^{-5}	346	173
10^{-6}	346	173

Table S3. Effect of T_{sel}; all single excitations were included and $T_{pre} = 10^{-4}$.

T_{sel}	*ΔE(S-T)* [cm^{-1}]	*J* [cm^{-1}]
10^{-4}	346	173
10^{-5}	346	173
10^{-6}	346	173
10^{-7}	346	173
10^{-8}	346	173
10^{-9}	346	173
10^{-10}	341	171

Table S4. Effect of T_{sel}; **not** all single excitations were included and $T_{pre} = 10^{-4}$.

T_{sel}	*ΔE(S-T)* [cm^{-1}]	*J* [cm^{-1}]
10^{-4}	642	321
10^{-5}	477	239
10^{-6}	364	182
10^{-7}	353	176
10^{-8}	346	173
10^{-9}	344	172
10^{-10}	344	172

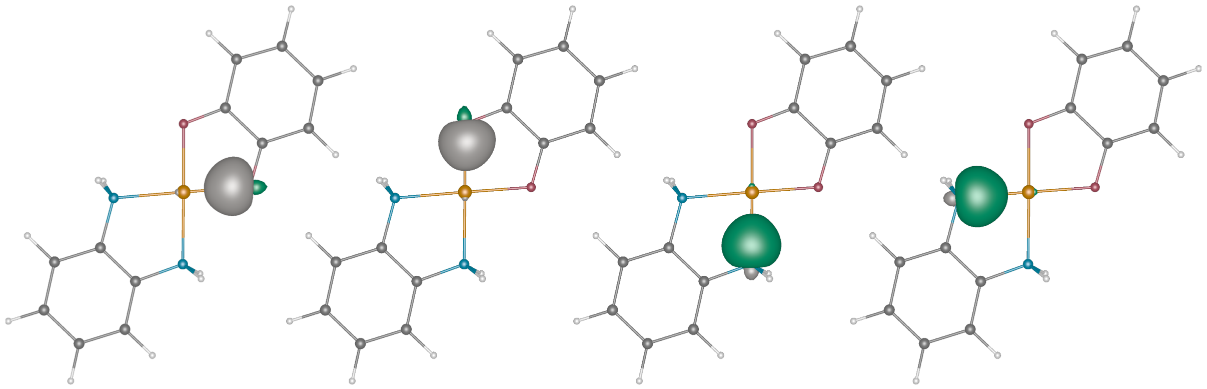

Figure S1. Lone pairs included in the (18e,10o) active space.

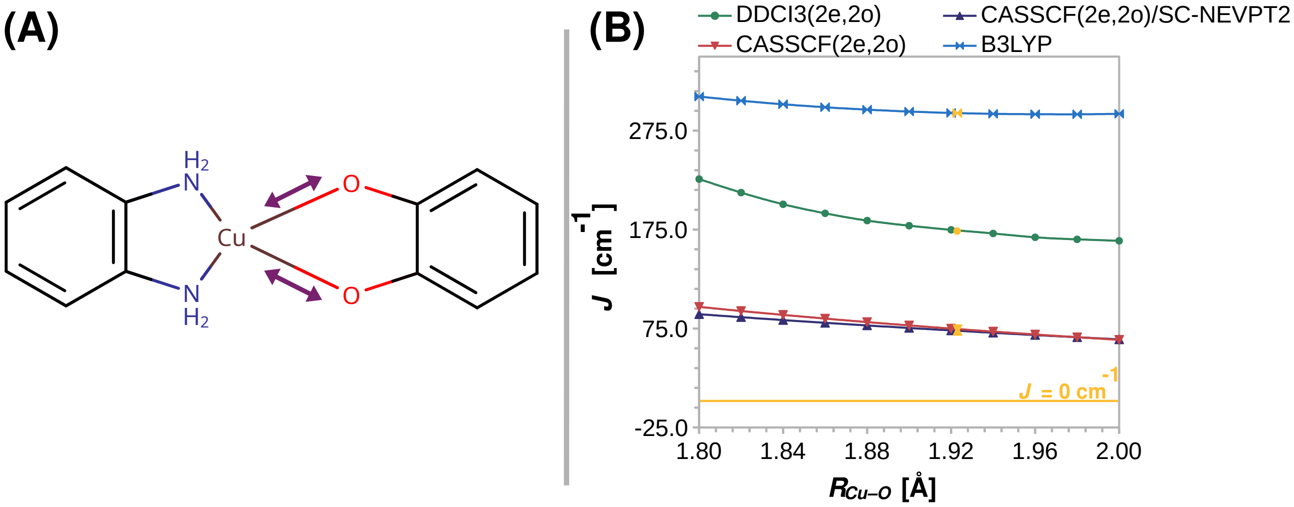

Figure S2. Rigid scan of J along the Cu–O distance ($R_{Cu\text{–}O}$): schematic representation of the varied structural parameter (A); the parameter J predicted at the B3LYP, CASSCF(2e,2o), CASSCF(2e,2o)/SC-NEVPT2 and DDCI3(2e,2o) levels (B). All are calculated with the def2-TZVP basis set. For clarity, the line J = 0 cm^{-1} is shown as a reference. For the geometry optimized at the B3LYP/def2-TZVP theory level $R_{Cu\text{–}O}$ = 1.925 Å (labeled with the yellow symbols).

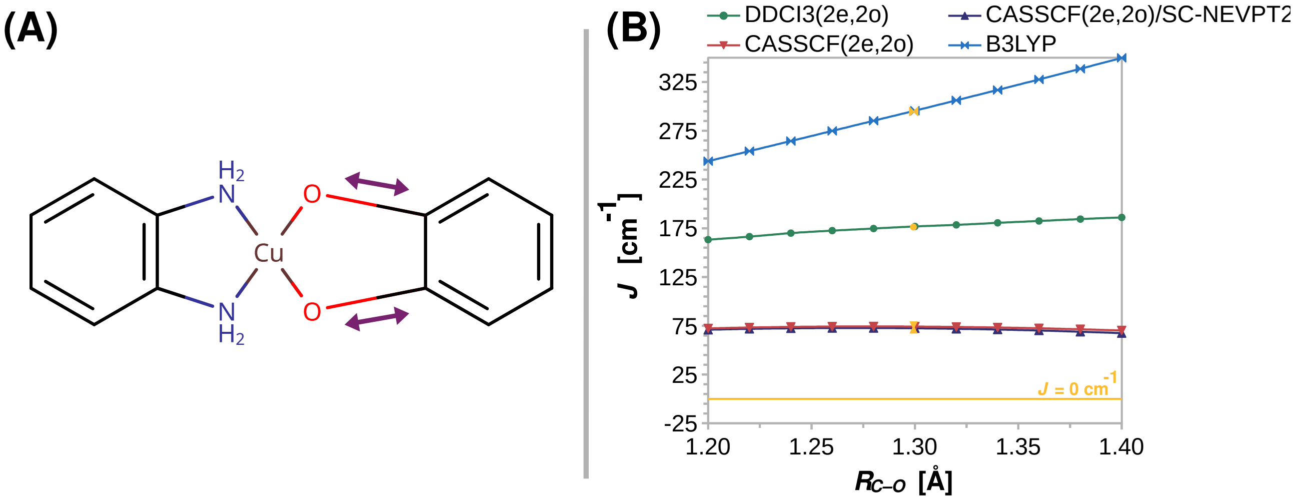

Figure S3. Rigid scan of J along the C–O distance ($R_{C\text{–}O}$): schematic representation of the varied structural parameter (A); the parameter J predicted at the B3LYP, CASSCF(2e,2o), CASSCF(2e,2o)/SC-NEVPT2 and DDCI3(2e,2o) levels (B). All are calculated with the def2-TZVP basis set. For clarity, the line J = 0 cm^{-1} is shown as a reference. For the geometry optimized at the B3LYP/def2-TZVP theory level $R_{C\text{–}O}$ = 1.294 Å (labeled with the yellow symbols).

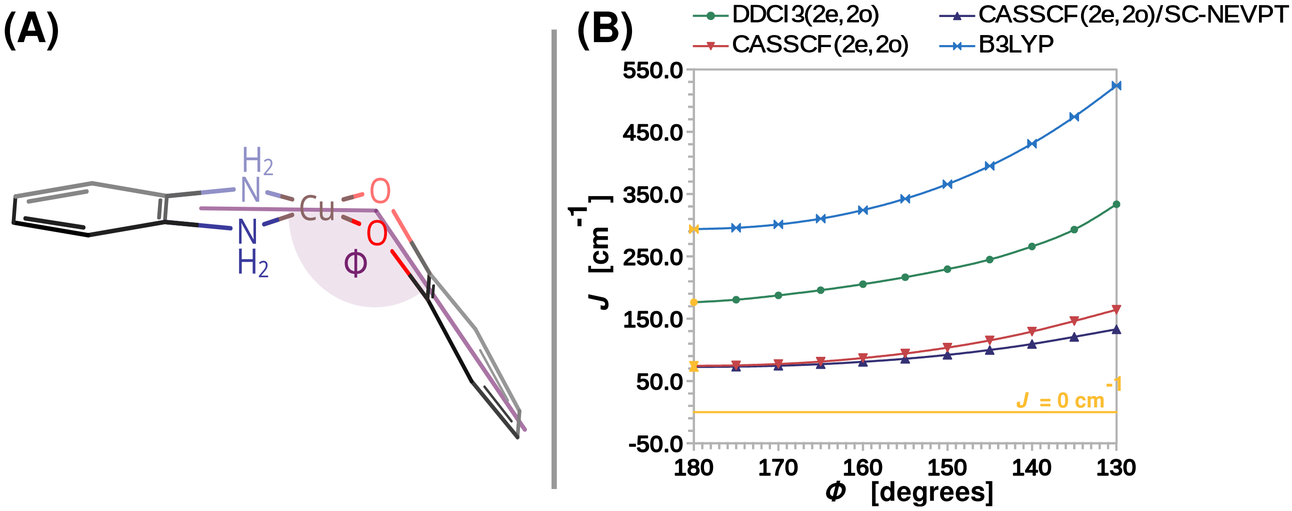

Figure S4. Rigid scan of *J* along the bending angle between the N-Cu-N and O-Cu-O planes (*Φ*): schematic representation of the varied structural parameter (A); the parameter *J* predicted at the B3LYP, CASSCF(2e,2o), CASSCF(2e,2o)/SC-NEVPT2 and DDCI3(2e,2o) levels (B). All are calculated with the def2-TZVP basis set. For clarity, the line $J = 0$ cm^{-1} is shown as a reference. For the geometry optimized at the B3LYP/def2-TZVP theory level Φ = 0° (labeled with the yellow symbols).

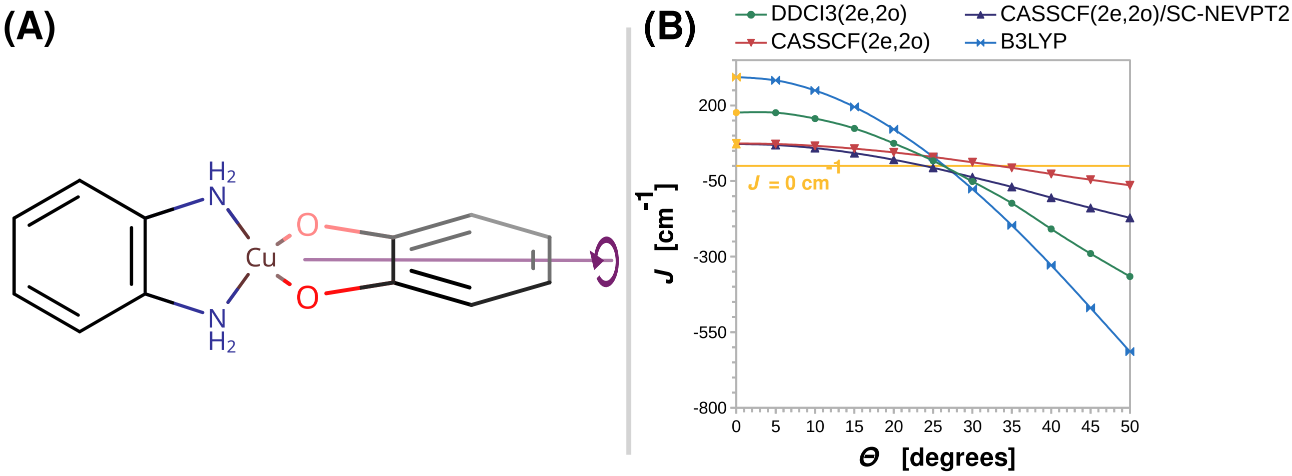

Figure S5. Rigid scan of *J* along the twisting angle between the N-Cu-N and O-Cu-O planes (*Θ*): schematic representation of the varied structural parameter (A); the parameter *J* predicted at the B3LYP, CASSCF(2e,2o), CASSCF(2e,2o)/SC-NEVPT2 and DDCI3(2e,2o) levels (B). All are calculated with the def2-TZVP basis set. For clarity, the line $J = 0$ cm^{-1} is shown as a reference. For the geometry optimized at the B3LYP/def2-TZVP theory level *Θ* = 0° (labeled with the yellow symbols).

Fully optimized structures in XYZ format. Coordinates given in Å.

$[Cu(SQ)(opd)]^+$

Cu	0.00000	0.00000	0.00000
O	1.91966	0.13654	0.00000
O	0.00000	1.92452	0.00000
C	1.21363	2.37456	-0.00001
C	2.28247	1.37904	-0.00001
C	1.53741	3.74718	-0.00002
C	3.62866	1.79939	-0.00002
C	2.85655	4.11362	-0.00004
C	3.90059	3.14120	-0.00004
H	4.41158	1.05402	-0.00002
H	0.73837	4.47525	-0.00002
H	3.12263	5.16193	-0.00005
H	4.92738	3.48099	-0.00005
N	-2.02765	-0.16684	0.00000
N	-0.02255	-2.03438	-0.00004
H	-2.39793	0.32180	0.81269
H	-2.39793	0.32184	-0.81267
H	0.49114	-2.36907	0.81263
H	0.49115	-2.36903	-0.81273
C	-1.39468	-2.51759	-0.00006
C	-2.41229	-1.56980	-0.00004
C	-1.70158	-3.87138	-0.00010
C	-3.74090	-1.97197	-0.00006
C	-3.02987	-4.27346	-0.00011
C	-4.04772	-3.32545	-0.00010
H	-4.52983	-1.23075	-0.00004
H	-0.90625	-4.60574	-0.00011
H	-3.27060	-5.32737	-0.00014
H	-5.08188	-3.64034	-0.00011

[Cu(SQ)(acac)]

O	1.95887	0.22415	0.00162
O	0.00000	1.97158	0.00000
C	1.19246	2.43668	-0.01746
C	2.28530	1.46179	-0.01632
C	1.49915	3.81985	-0.03916
C	3.62456	1.92395	-0.03665
C	2.80916	4.21812	-0.06001
C	3.87115	3.27077	-0.05868
H	4.42271	1.19405	-0.03575
H	0.68309	4.52966	-0.04019
H	3.05058	5.27292	-0.07796
H	4.89166	3.63064	-0.07565
O	-1.91476	-0.04462	-0.00671
O	0.17233	-1.90764	-0.01426
C	-0.78854	-2.73897	-0.03556
C	-2.63179	-1.09360	-0.02842
C	-0.37486	-4.18598	-0.05417
C	-4.11648	-0.84653	-0.03793
H	-4.69061	-1.77019	-0.06032
H	-4.37092	-0.23829	-0.90762
H	-4.38796	-0.27011	0.84811
H	-1.22769	-4.86115	-0.06488
H	0.24264	-4.39203	0.82168
H	0.24413	-4.36801	-0.93445
C	-2.14670	-2.40522	-0.04353
H	-2.86505	-3.20987	-0.06215

[Cu(SQ)(dtc)]

Cu	0.00000	0.00000	0.00000
O	1.97588	0.22050	0.04856
O	0.00000	1.98818	0.00000
C	1.19664	2.43924	0.03022
C	2.29119	1.46012	0.05604
C	1.51103	3.82221	0.03997
C	3.63025	1.92647	0.08742
C	2.82034	4.22076	0.07184

C	3.88062	3.27235	0.09435
H	4.42688	1.19490	0.10485
H	0.69564	4.53278	0.02200
H	3.06247	5.27558	0.07974
H	4.90168	3.63042	0.11785
S	-2.27972	-0.39746	-0.08397
S	-0.12493	-2.31110	0.02515
C	-1.82583	-2.05731	-0.04548
N	-2.70897	-3.05244	-0.06839
C	-4.14326	-2.82484	-0.17378
C	-2.31581	-4.45181	0.01630
H	-4.33802	-1.79454	-0.45284
H	-4.63173	-3.03760	0.78015
H	-4.55451	-3.48989	-0.93473
H	-1.26987	-4.52926	0.29410
H	-2.47045	-4.94661	-0.94544
H	-2.92810	-4.95001	0.76975

$[Cu(SQ)(en)]^{+}$

Cu	0.00000	0.00000	0.00000
O	1.91960	0.13669	-0.00267
O	0.00000	1.92423	0.00000
C	1.21366	2.37456	-0.00543
C	2.28259	1.37918	-0.00435
C	1.53707	3.74726	-0.01222
C	3.62881	1.79942	-0.00527
C	2.85620	4.11367	-0.01456
C	3.90044	3.14126	-0.01044
H	4.41188	1.05421	-0.00264
H	0.73797	4.47524	-0.01503
H	3.12231	5.16196	-0.01924
H	4.92717	3.48123	-0.01192
N	-2.03578	-0.17170	0.01746
N	-0.02100	-2.04318	-0.01872
H	-2.37649	0.07940	0.94257
H	-2.44685	0.49846	-0.62590
H	0.67599	-2.40367	0.62644
H	0.25746	-2.36374	-0.94318
C	-1.38908	-2.50573	0.32354
C	-2.39542	-1.56923	-0.32960
H	-3.41060	-1.81773	-0.01927
H	-2.34710	-1.66138	-1.41533
H	-1.56121	-3.53559	0.00979
H	-1.48771	-2.46756	1.40909

$[Cu(SQ)(mnt)]^{-}$

Cu	0.00000	0.00000	0.00000
O	2.00080	0.28770	0.00010
O	0.00000	2.02136	0.00000
C	1.18805	2.48999	0.00019
C	2.29555	1.53036	0.00025
C	1.48510	3.87716	0.00038
C	3.62629	2.02186	0.00045
C	2.79041	4.30258	0.00058
C	3.86157	3.37444	0.00063
H	4.43452	1.30193	0.00048
H	0.65744	4.57467	0.00035
H	3.01182	5.36281	0.00072
H	4.87949	3.74452	0.00079
S	-2.24910	-0.10955	0.00058
S	0.21139	-2.24182	-0.00062
C	-1.45683	-2.72937	-0.00017
C	-2.49446	-1.83017	0.00034
C	-1.70360	-4.12806	-0.00031
C	-3.84383	-2.27334	0.00070
N	-1.87114	-5.27154	-0.00066
N	-4.95182	-2.60192	0.00105

[Cu(SQ)(opd)(HCOO)]

Cu	0.00000	0.00000	0.00000

O	1.96056	0.19808	0.01676
O	0.00000	1.97167	0.00000
C	1.19463	2.41454	0.14237
C	2.28133	1.43208	0.14932
C	1.50682	3.78931	0.28312
C	3.61662	1.88290	0.29322
C	2.81320	4.17598	0.42061
C	3.86843	3.22252	0.42544
H	4.40980	1.14756	0.30162
H	0.69469	4.50364	0.28301
H	3.05490	5.22478	0.53320
H	4.88681	3.56976	0.54155
N	-1.96795	-0.21402	-0.49444
N	0.02058	-2.03954	-0.41275
H	-2.32530	-0.31901	0.51231
H	-2.40715	0.59196	-0.92337
H	-0.05271	-2.33995	0.55835
H	0.87915	-2.40674	-0.80558
C	-1.14054	-2.36746	-1.20123
C	-2.15336	-1.40733	-1.25629
C	-1.24843	-3.54430	-1.92767
C	-3.27344	-1.64309	-2.04439
C	-2.36690	-3.77024	-2.72079
C	-3.37768	-2.81845	-2.77746
H	-4.06064	-0.90095	-2.08318
H	-0.45327	-4.27791	-1.87886
H	-2.44640	-4.68531	-3.29158
H	-4.25070	-2.99009	-3.39263
O	-0.29035	-0.63759	2.08573
C	-1.42836	-0.66575	2.63187
O	-2.54005	-0.54106	2.07456
H	-1.43390	-0.82849	3.72718

[Cu(SQ)(opd)(Cl)]

Cu	0.00000	0.00000	0.00000
O	1.95273	0.19170	0.02296
O	0.00000	1.96277	0.00000
C	1.19461	2.40793	0.15079
C	2.27762	1.42565	0.16354
C	1.50588	3.78128	0.29678
C	3.61220	1.87082	0.32169
C	2.81227	4.16515	0.44667
C	3.86529	3.21003	0.45918
H	4.40349	1.13369	0.33680
H	0.69523	4.49722	0.29280
H	3.05459	5.21323	0.56442
H	4.88322	3.55459	0.58625
N	-2.00208	-0.20267	-0.56260
N	-0.00044	-2.01746	-0.55170
H	-2.28074	-0.39747	0.40707
H	-2.48134	0.62136	-0.90427
H	-0.17293	-2.30717	0.41893
H	0.86835	-2.41641	-0.88552
C	-1.11248	-2.27815	-1.41778
C	-2.14752	-1.33952	-1.42369
C	-1.17044	-3.37618	-2.26422
C	-3.22929	-1.50858	-2.27608
C	-2.25082	-3.53751	-3.12365
C	-3.27947	-2.60440	-3.12963
H	-4.02743	-0.77690	-2.27616
H	-0.36472	-4.09945	-2.25504
H	-2.28641	-4.39126	-3.78652
H	-4.12145	-2.72653	-3.79723
Cl	-0.80877	-0.88013	2.18464

$[Cu(SQ)(opd)(NH_3)]^+$

Cu	0.00000	0.00000	0.00000
O	1.94593	0.17317	0.00416
O	0.00000	1.95351	0.00000
C	1.20959	2.40467	0.02083

C	2.28793	1.41810	0.02253
C	1.52688	3.78122	0.04466
C	3.63072	1.85679	0.04640
C	2.84175	4.16210	0.06482
C	3.89305	3.20040	0.06550
H	4.42059	1.11839	0.04600
H	0.72102	4.50214	0.04300
H	3.09686	5.21312	0.08074
H	4.91718	3.54801	0.08182
N	-1.99395	-0.16365	-0.53344
N	0.01377	-2.00203	-0.53130
H	-2.57382	0.42513	0.05656
H	-2.09955	0.19008	-1.48189
H	0.65029	-2.52662	0.06086
H	0.37704	-2.07783	-1.47897
C	-1.34372	-2.50042	-0.43123
C	-2.37116	-1.55953	-0.43205
C	-1.63681	-3.85093	-0.30460
C	-3.69085	-1.96973	-0.30583
C	-2.95767	-4.26010	-0.17264
C	-3.98274	-3.32126	-0.17323
H	-4.48636	-1.23539	-0.30855
H	-0.83558	-4.57901	-0.30624
H	-3.18527	-5.31202	-0.07071
H	-5.01073	-3.64011	-0.07178
N	-0.23285	-0.24443	2.26566
H	-0.49805	0.66678	2.62761
H	-0.86878	-0.92482	2.66935
H	0.69281	-0.44299	2.63355

[Cu(SQ)(opd)(H2O)]⁺

Cu	0.00000	0.00000	0.00000
O	1.93308	0.15940	0.00020
O	0.00000	1.94692	0.00000
C	1.21328	2.39475	-0.04114
C	2.28536	1.40051	-0.04009
C	1.53701	3.76753	-0.08174
C	3.63137	1.82871	-0.08003
C	2.85480	4.13855	-0.12291
C	3.90100	3.16977	-0.12193
H	4.41602	1.08500	-0.07964
H	0.73671	4.49457	-0.08559
H	3.11601	5.18756	-0.15828
H	4.92676	3.51109	-0.15592
N	-2.01319	-0.20357	-0.26136
N	0.01177	-2.03331	-0.15827
H	-2.38507	-0.00234	0.66594
H	-2.39909	0.48410	-0.90168
H	0.24701	-2.42929	0.74905
H	0.75587	-2.31197	-0.79324
C	-1.28767	-2.49393	-0.62047
C	-2.31630	-1.55883	-0.67706
C	-1.51204	-3.80493	-1.01684
C	-3.57247	-1.93346	-1.13372
C	-2.76813	-4.17807	-1.47561
C	-3.79611	-3.24338	-1.53480
H	-4.36996	-1.20277	-1.17725
H	-0.70712	-4.52756	-0.97358
H	-2.94301	-5.19755	-1.78982
H	-4.77338	-3.53409	-1.89420
O	-0.75290	0.32887	2.34376
H	-0.52574	-0.00734	3.21867
H	-0.61565	1.28472	2.37842

[Cu(SQ)(opd)(ClO_4)]

Cu	0.00000	0.00000	0.00000
O	1.95164	0.17858	0.01639
O	0.00000	1.95969	0.00000
C	1.19490	2.39728	0.16858
C	2.27683	1.41008	0.17607

C	1.50811	3.76748	0.33745
C	3.61135	1.84947	0.34913
C	2.81353	4.14409	0.50440
C	3.86504	3.18504	0.50977
H	4.40060	1.11025	0.35949
H	0.69895	4.48491	0.33950
H	3.05837	5.18898	0.64224
H	4.88219	3.52586	0.65123
N	-2.00549	-0.20401	-0.36429
N	-0.02510	-2.02284	-0.32585
H	-2.38427	-0.33411	0.58807
H	-2.43018	0.61868	-0.77666
H	-0.14602	-2.39191	0.63127
H	0.83842	-2.38617	-0.71254
C	-1.16883	-2.32813	-1.14616
C	-2.19518	-1.38473	-1.16654
C	-1.26966	-3.48161	-1.90996
C	-3.31830	-1.59719	-1.95242
C	-2.39160	-3.68946	-2.70294
C	-3.41377	-2.74845	-2.72464
H	-4.11309	-0.86220	-1.96137
H	-0.47114	-4.21217	-1.88527
H	-2.46638	-4.58685	-3.30176
H	-4.28775	-2.90995	-3.34075
O	-0.22811	-0.21693	2.28979
Cl	-1.17028	-1.20600	2.84350
O	-1.14684	-1.16417	4.26786
O	-0.77906	-2.52797	2.35368
O	-2.50619	-0.89517	2.33433

$[Cu(SQ)(opd)(THF)]^+$

Cu	0.00000	0.00000	0.00000
O	1.94196	0.16294	0.00901
O	0.00000	1.94827	0.00000
C	1.20887	2.39525	0.06207
C	2.28560	1.40494	0.06744
C	1.52910	3.76982	0.12657
C	3.62825	1.83971	0.13935
C	2.84379	4.14641	0.19175
C	3.89281	3.18158	0.19896
H	4.41644	1.09957	0.14273
H	0.72557	4.49331	0.11881
H	3.10068	5.19610	0.23852
H	4.91685	3.52582	0.25222
N	-2.00854	-0.16591	-0.37035
N	-0.01266	-2.02632	-0.29366
H	-2.49020	-0.06250	0.51979
H	-2.32214	0.59531	-0.96623
H	0.07403	-2.44329	0.63053
H	0.79252	-2.32658	-0.83575
C	-1.26003	-2.40943	-0.92814
C	-2.27717	-1.46104	-0.96839
C	-1.44896	-3.65937	-1.50094
C	-3.48454	-1.76008	-1.58397
C	-2.65529	-3.95688	-2.12037
C	-3.67108	-3.00840	-2.16250
H	-4.27152	-1.01745	-1.61715
H	-0.65355	-4.39299	-1.46834
H	-2.80040	-4.92804	-2.57258
H	-4.60883	-3.23907	-2.64840
O	-0.22835	-0.28855	2.28273
C	-0.87575	0.79185	3.01293
C	0.10213	1.22802	4.11108
H	-1.81758	0.41960	3.41875
H	-1.07616	1.58836	2.29668
C	1.43247	0.59525	3.68373
H	-0.20973	0.83561	5.07881
H	0.16028	2.31154	4.19292
C	0.97223	-0.67454	2.99386
H	2.09525	0.39708	4.52423

H	1.96057	1.23428	2.97448
H	0.72279	-1.45886	3.71444
H	1.68088	-1.05776	2.26204

[Cu(SQ)(opd)(BF4)]

Cu	0.00000	0.00000	0.00000
O	1.94961	0.17686	0.01575
O	0.00000	1.95796	0.00000
C	1.19856	2.39939	0.12839
C	2.28031	1.41187	0.13351
C	1.51598	3.77319	0.25583
C	3.61921	1.85406	0.25828
C	2.82542	4.15255	0.37793
C	3.87701	3.19310	0.37762
H	4.40869	1.11503	0.26393
H	0.70736	4.49117	0.26126
H	3.07416	5.20035	0.48273
H	4.89777	3.53692	0.48051
N	-2.00595	-0.21818	-0.34399
N	-0.02331	-2.02468	-0.29597
H	-2.38064	-0.36945	0.60535
H	-2.44522	0.60395	-0.74195
H	-0.16987	-2.38329	0.65993
H	0.84625	-2.39757	-0.65928
C	-1.14930	-2.32659	-1.14236
C	-2.17987	-1.38796	-1.16674
C	-1.22720	-3.46560	-1.92925
C	-3.28608	-1.59143	-1.97757
C	-2.33254	-3.66456	-2.74809
C	-3.35969	-2.72941	-2.77218
H	-4.08446	-0.86040	-1.98917
H	-0.42462	-4.19168	-1.90326
H	-2.38991	-4.55062	-3.36535
H	-4.22060	-2.88442	-3.40798
F	-0.34337	-0.28109	2.25707
B	-1.25175	-1.25446	2.79857
F	-1.24715	-1.22726	4.15844
F	-0.84628	-2.51825	2.29081
F	-2.53587	-0.96143	2.26389

Structures determined from X-ray diffraction with the optimized positions of hydrogen atoms. XYZ coordinates given in Å.

[Cu(dtbSQ)(dpya)(ClO_4)]

Cu	0.00000	0.00000	0.00000
Cl	1.16812	-1.16508	3.19911
O	1.93006	0.25430	-0.23413
O	0.00000	1.96427	0.00000
O	0.71546	-1.08012	4.59814
O	0.35624	-0.20863	2.41318
O	1.06996	-2.54101	2.87502
O	2.52033	-0.76430	3.13406
N	0.05932	-1.91409	-0.55828
N	-2.29172	-2.18822	-0.60805
N	-1.93177	-0.07872	0.38928
C	2.26771	1.50026	-0.31982
C	3.60660	1.94873	-0.51500
C	3.76563	3.29353	-0.66393
C	2.70159	4.26471	-0.62932
C	1.43149	3.83022	-0.39710
C	1.18524	2.45618	-0.22911
C	3.06210	5.72493	-0.86594
C	3.57661	5.87147	-2.31065
C	4.16914	6.16905	0.10003
C	1.85630	6.65469	-0.68851
C	4.77626	0.96654	-0.54296
C	4.82778	0.19942	0.77962
C	4.59920	-0.01877	-1.71447
C	6.10734	1.67572	-0.74121
C	1.27229	-2.43193	-0.84338
C	1.44658	-3.61153	-1.51846
C	0.33781	-4.31574	-1.92900
C	-0.90134	-3.83022	-1.61578
C	-1.00894	-2.61728	-0.91763
C	-2.76395	-1.05145	-0.00155
C	-4.16096	-0.95628	0.17165
C	-4.67025	0.12998	0.81156
C	-3.83243	1.12107	1.28467
C	-2.49249	0.98116	1.03786
H	-3.01066	-2.83410	-0.88569
H	4.76022	3.67411	-0.82983
H	0.58723	4.50031	-0.33688
H	3.85014	6.91020	-2.50717
H	2.80823	5.57884	-3.02831
H	4.45653	5.25299	-2.49037
H	4.41273	7.21863	-0.07599
H	5.08482	5.59311	-0.02766
H	3.84909	6.06463	1.13807
H	2.16168	7.68710	-0.86393
H	1.44743	6.59341	0.32113
H	1.05968	6.42141	-1.39647
H	5.65473	-0.51422	0.76136
H	3.90985	-0.34211	0.99044
H	4.99259	0.88071	1.61600
H	5.42897	-0.72857	-1.72557
H	4.59347	0.51053	-2.66997
H	3.67041	-0.57657	-1.62968
H	6.91006	0.93701	-0.74382
H	6.31766	2.38094	0.06523
H	6.15317	2.21410	-1.69052
H	2.10837	-1.84399	-0.50252
H	2.44853	-3.96495	-1.71215
H	0.43613	-5.24563	-2.47317
H	-1.80028	-4.36077	-1.90025
H	-4.79846	-1.75282	-0.18696
H	-5.74081	0.20636	0.95676
H	-4.19877	1.98603	1.81523
H	-1.78015	1.72928	1.34940

[Cu(dtbSQ)(tmcd)(THF)]⁺

Cu	0.00000	0.00000	0.00000
O	1.93824	0.20482	-0.01086
O	0.00000	1.96249	0.00000
C	2.26949	1.44940	0.08005
C	1.19745	2.43267	0.08282
C	3.61467	1.86740	0.15617
C	1.51630	3.82827	0.17792
C	3.91789	3.20201	0.21237
C	2.84446	4.14631	0.22568
C	0.39881	4.88136	0.22020
C	-0.46870	4.64990	1.47066
C	0.95955	6.30939	0.31670
C	-0.44648	4.78685	-1.05682
C	5.35576	3.70964	0.20419
C	5.54189	4.78760	1.27246
C	6.36110	2.63235	0.43503
C	5.60096	4.35366	-1.15904
H	3.11109	5.18898	0.27612
H	4.37466	1.10097	0.14706
H	-0.92652	3.66272	1.45906
H	-1.26388	5.39578	1.51733
H	0.13360	4.74350	2.37589
H	1.57649	6.56519	-0.54616
H	1.55099	6.45375	1.22216
H	0.13058	7.01668	0.34883
H	-0.90830	3.80813	-1.15823
H	0.16891	4.96977	-1.93916
H	-1.23690	5.53947	-1.03760
H	5.31567	4.40375	2.26938
H	4.91978	5.66436	1.09990
H	6.57912	5.12553	1.27369
H	6.21306	2.13176	1.39443
H	7.36584	3.05656	0.44067
H	6.34359	1.87174	-0.34772
H	5.50174	3.62291	-1.96376
H	6.60994	4.76821	-1.20217
H	4.89965	5.16506	-1.35573
N	0.05850	-1.99607	-0.15335
N	-1.94522	-0.11499	-0.45541
C	-1.37259	-2.45110	-0.10280
C	-2.25268	-1.54569	-0.77375
C	-1.59871	-3.91340	-0.49043
C	-3.71433	-1.92442	-0.70987
C	-3.06082	-4.28309	-0.43911
C	-3.95942	-3.36580	-1.03335
C	0.76442	-2.58925	1.01990
C	0.80410	-2.34156	-1.37534
C	-2.73684	0.45921	0.64922
C	-2.21837	0.69217	-1.66801
H	-1.60443	-2.39121	0.97026
H	-1.99397	-1.61346	-1.84109
H	-1.02115	-4.57205	0.15448
H	-1.22872	-4.06253	-1.51057
H	-4.07545	-1.73379	0.30733
H	-4.29947	-1.28161	-1.36640
H	-3.31578	-4.40660	0.62477
H	-3.18209	-5.28416	-0.86021
H	-4.99534	-3.61104	-0.78823
H	-3.90997	-3.46362	-2.12868
H	0.78508	-3.67729	0.94712
H	0.26281	-2.28677	1.93322
H	1.78544	-2.21721	1.03256
H	0.97111	-3.41749	-1.44997
H	1.76259	-1.83043	-1.34446
H	0.26006	-2.00951	-2.25883
H	-3.79396	0.52422	0.38849
H	-2.36142	1.45863	0.84594
H	-2.62568	-0.14451	1.54695
H	-3.26477	0.60243	-1.96583

H	-1.58284	0.35966	-2.48602
H	-1.99522	1.73390	-1.45750
O	0.16740	0.22245	2.45602
C	1.37025	-0.02745	3.18500
C	-0.65000	1.00579	3.32079
C	1.62660	1.23325	3.95409
C	0.25036	1.75365	4.27595
H	1.21562	-0.88299	3.85560
H	2.14859	-0.27880	2.46963
H	-1.33956	0.34302	3.85563
H	-1.23909	1.67224	2.69573
H	2.24070	1.05926	4.83693
H	2.16560	1.93837	3.31764
H	0.18100	2.83084	4.13685
H	-0.02854	1.54484	5.30858

[Cu(dtbSQ)(bipy)(BF_4)]

Cu	0.00000	0.00000	0.00000
O	1.92555	0.16442	-0.03945
O	0.00000	1.93589	0.00000
N	0.02799	-1.94536	0.27658
N	-1.90454	-0.23920	0.42426
F	-0.15834	0.31937	-2.32574
F	0.35158	0.20955	-4.49805
F	1.30157	-1.22063	-3.11206
F	-0.88431	-1.37926	-3.59040
B	0.11718	-0.53232	-3.38255
C	1.09377	-2.74981	0.14361
H	1.98268	-2.27320	-0.24391
C	1.04451	-4.08985	0.45371
H	1.92073	-4.70445	0.30853
C	-0.13862	-4.61976	0.94375
H	-0.20665	-5.66805	1.20111
C	-1.23122	-3.79549	1.10273
H	-2.16232	-4.18977	1.48143
C	-1.13316	-2.45613	0.73930
C	-2.24635	-1.48669	0.81131
C	-3.53003	-1.78149	1.21016
H	-3.79092	-2.78163	1.52233
C	-4.48238	-0.79631	1.17561
H	-5.49917	-1.02067	1.47023
C	-4.15120	0.48071	0.76057
H	-4.88529	1.27159	0.71763
C	-2.83071	0.73682	0.39702
H	-2.47727	1.70628	0.07497
C	2.27665	1.40364	-0.17273
C	1.19781	2.37610	-0.16240
C	1.49518	3.74479	-0.37063
H	0.66099	4.42726	-0.40122
C	2.78817	4.15089	-0.55865
C	3.84646	3.17226	-0.50699
H	4.85619	3.52586	-0.64096
C	3.63508	1.82528	-0.32799
C	3.13317	5.61039	-0.84228
C	1.89213	6.49331	-0.89635
H	1.20793	6.17929	-1.68540
H	2.18810	7.52236	-1.10579
H	1.34916	6.49347	0.04993
C	4.06680	6.14121	0.25515
H	4.32221	7.18400	0.05636
H	4.99688	5.57463	0.30575
H	3.58795	6.08877	1.23451
C	3.84558	5.71366	-2.18999
H	4.77424	5.14264	-2.20733
H	4.09286	6.75530	-2.40440
H	3.21272	5.34112	-2.99696
C	4.76256	0.79568	-0.30724
C	4.77201	0.06531	1.05278
H	4.94407	0.76797	1.87118
H	5.57588	-0.67363	1.06786

H	3.82923	-0.44645	1.23095
C	4.53606	-0.24753	-1.42995
H	3.57066	-0.73589	-1.35050
H	5.32385	-1.00298	-1.38736
H	4.57615	0.22764	-2.41115
C	6.13895	1.43673	-0.52027
H	6.20407	1.94103	-1.48575
H	6.90340	0.65900	-0.50208
H	6.37989	2.15670	0.26473

$[Cu(dtbSQ)(dpya)(THF)_2]^+$

Cu	0.00000	0.00000	0.00000
O	1.95716	0.24301	-0.13690
O	0.00000	1.97824	0.00000
O	0.48792	0.21534	2.45161
O	-0.26183	-0.04646	-2.26386
N	0.17482	-1.95185	0.21776
N	-1.60564	-2.02765	1.75483
H	-2.03406	-2.59805	2.46530
N	-1.92361	-0.11093	0.41096
C	1.19596	-2.58991	-0.40688
H	1.72429	-1.99301	-1.13496
C	1.55825	-3.87263	-0.10243
H	2.38161	-4.33874	-0.62296
C	0.87452	-4.53228	0.90309
H	1.15660	-5.53700	1.18837
C	-0.17563	-3.91735	1.53224
H	-0.74149	-4.42204	2.30364
C	-0.53008	-2.61772	1.14159
C	-2.40201	-0.96102	1.34216
C	-3.64784	-0.78714	1.91682
H	-3.97432	-1.46446	2.69557
C	-4.47736	0.21444	1.47087
H	-5.45682	0.35188	1.90639
C	-4.02572	1.03486	0.43202
H	-4.64745	1.82236	0.03225
C	-2.77003	0.85219	-0.05358
H	-2.34562	1.49671	-0.80857
C	6.37900	2.62572	0.63250
H	6.38496	2.18698	-0.36636
H	6.23102	1.82634	1.36014
H	7.36098	3.06184	0.81198
C	5.65573	4.82611	-0.23303
H	5.61619	4.45582	-1.25898
H	6.66749	5.18607	-0.04266
H	4.98343	5.68170	-0.16216
C	5.39725	4.30045	2.18772
H	4.70337	5.12714	2.34187
H	6.40601	4.67412	2.36793
H	5.17889	3.53447	2.93368
C	5.30098	3.72493	0.76322
C	3.90696	3.21047	0.52008
C	3.59708	1.89071	0.28055
H	4.36552	1.13235	0.22659
C	2.29056	1.47592	0.09314
C	1.18367	2.42772	0.15742
C	1.50928	3.82651	0.41372
C	2.79686	4.13747	0.57903
H	3.05262	5.16504	0.78711
C	-0.62187	4.43522	1.62967
H	-1.08974	3.47637	1.42089
H	-1.40616	5.18846	1.72632
H	-0.10581	4.36885	2.58983
C	0.35139	4.84131	0.51936
C	-0.38162	4.92429	-0.82538
H	-0.80164	3.96132	-1.10951
H	0.29669	5.25094	-1.61530
H	-1.19638	5.64844	-0.76228
C	0.89553	6.24657	0.84877
H	1.57142	6.61158	0.07462

H	1.41991	6.26381	1.80541
H	0.06334	6.94812	0.91646
C	1.54347	-0.53348	3.05626
H	1.33223	-1.59553	2.86622
H	2.44945	-0.31386	2.48687
C	1.59574	-0.21952	4.37928
H	2.52493	0.31315	4.61275
H	1.66887	-1.11517	5.00424
C	0.47750	0.59411	4.78151
H	0.77975	1.51890	5.27108
H	-0.18662	0.08789	5.48393
C	-0.25615	0.88135	3.48814
H	-1.27839	0.49199	3.49236
H	-0.30414	1.94059	3.23704
C	-1.05014	-0.96490	-3.03304
H	-2.10540	-0.67438	-2.94296
H	-0.94185	-1.95185	-2.58329
C	-0.55421	-0.85585	-4.38618
H	-1.34272	-1.01446	-5.12351
H	0.17615	-1.64862	-4.57368
C	0.09530	0.46212	-4.52368
H	-0.51798	1.15723	-5.09931
H	1.04787	0.39296	-5.04655
C	0.26094	0.95634	-3.12177
H	1.30016	1.11699	-2.83335
H	-0.27559	1.89257	-2.94322

[Cu(dtbSQ)(dtben)]$^{+}$

Cu	0.00000	0.00000	0.00000
O	1.83675	0.23641	-0.52155
O	0.00000	1.97508	0.00000
C	2.11476	1.44395	-0.79329
C	1.13358	2.44769	-0.37743
C	3.29615	1.85790	-1.46632
C	1.45709	3.84923	-0.46924
C	3.58298	3.17587	-1.59501
C	2.64177	4.14833	-1.07894
C	0.48226	4.91480	0.01441
C	1.04367	6.33019	-0.21101
C	0.22171	4.73594	1.50560
C	-0.83417	4.79126	-0.76568
C	4.84354	3.70214	-2.28492
C	4.41559	4.58505	-3.47535
C	5.71483	2.57458	-2.82589
C	5.65298	4.53270	-1.27807
H	2.91422	5.18458	-1.18760
H	3.96603	1.08344	-1.80986
H	-0.22198	3.76572	1.71827
H	-0.46798	5.50414	1.86009
H	1.14605	4.82553	2.07934
H	1.20888	6.53875	-1.26880
H	1.98002	6.48672	0.32683
H	0.32520	7.06163	0.15944
H	-1.30897	3.82744	-0.59962
H	-0.66180	4.91224	-1.83673
H	-1.52650	5.57352	-0.44879
H	3.83036	4.00886	-4.19341
H	5.30063	4.96712	-3.98643
H	3.81957	5.44158	-3.16178
H	6.07408	1.91869	-2.03108
H	6.58980	2.99578	-3.32150
H	5.18134	1.96795	-3.55925
H	5.95373	3.92501	-0.42320
H	5.09043	5.38733	-0.90296
H	6.55587	4.91526	-1.75666
N	-0.52544	-1.56320	-1.11975
N	-1.34197	-0.57966	1.36680
C	-1.75750	-2.10351	-0.48662
C	-1.61923	-1.99997	1.01074
C	-0.62845	-1.34216	-2.61193

C	-1.33558	-2.51377	-3.27497
C	0.79298	-1.25894	-3.14970
C	-1.39532	-0.05144	-2.87081
C	-1.00950	-0.34094	2.82249
C	0.42482	-0.79693	3.06673
C	-1.15216	1.14346	3.06348
C	-1.98045	-1.11568	3.72324
H	0.21066	-2.25618	-0.99013
H	-2.19465	-0.05222	1.18263
H	-1.94122	-3.13674	-0.77878
H	-2.60422	-1.50838	-0.82764
H	-2.52320	-2.36480	1.49696
H	-0.78553	-2.60788	1.36048
H	1.32858	-2.19592	-2.97900
H	1.36041	-0.45800	-2.68434
H	0.76651	-1.08735	-4.22488
H	-0.85361	-3.46342	-3.03275
H	-1.28875	-2.39290	-4.35667
H	-2.38921	-2.58004	-3.00540
H	-1.47679	0.11580	-3.94405
H	-0.88609	0.81270	-2.44013
H	-2.41042	-0.08766	-2.47317
H	-3.01940	-0.87784	3.48498
H	-1.80915	-0.82745	4.75987
H	-1.84197	-2.19393	3.65873
H	-2.17949	1.47598	2.89466
H	-0.49883	1.71696	2.40853
H	-0.89631	1.38147	4.09502
H	0.55762	-1.86277	2.87988
H	0.69465	-0.61706	4.10668
H	1.13028	-0.24315	2.44307
